# The Application of and Strategy for Gold Nanoparticles in *Cancer* Immunotherapy

**DOI:** 10.3389/fphar.2021.687399

**Published:** 2021-06-07

**Authors:** Jia-shuai He, Shi-jin Liu, Yi-ran Zhang, Xiao-dong Chu, Zheng-bin Lin, Zhan Zhao, Sheng-hui Qiu, Yan-guan Guo, Hui Ding, Yun-long Pan, Jing-hua Pan

**Affiliations:** Department of General Surgery, The First Affiliated Hospital of Jinan University, Guangzhou, China

**Keywords:** nanomaterials, gold nanoparticles, cancer immunotherapy, nanocarriers, drug delivery system

## Abstract

Immunotherapy of malignant tumor is a verified and crucial anti-tumor strategy to help patients with cancer for prolonging prognostic survival. It is a novel anticancer tactics that activates the immune system to discern and damage cancer cells, thereby prevent them from proliferating. However, immunotherapy still faces many challenges in view of clinical efficacy and safety issues. Various nanomaterials, especially gold nanoparticles (AuNPs), have been developed not only for anticancer treatment but also for delivering antitumor drugs or combining other treatment strategies. Recently, some studies have focused on AuNPs for enhancing cancer immunotherapy. In this review, we summarized how AuNPs applicated as immune agents, drug carriers or combinations with other immunotherapies for anticancer treatment. AuNPs can not only act as immune regulators but also deliver immune drugs for cancer. Therefore, AuNPs are candidates for enhancing the efficiency and safety of cancer immunotherapy.

## Introduction of Immunotherapy and Nanoparticles

*Cancer* immunotherapy has rapidly emerged in the past few years (Hegde and Chen, 2020). *Cancer* immunotherapy has been applied in sorts of cancers such as melanoma, nonsmall cell lung cancer and colorectal cancer. Most cancer patients who received immunotherapy gained a great breakthrough therapeutic effect and prolonged prognosis compared with those who underwent traditional chemotherapy and radiotherapy. *Cancer* immunotherapy at present mainly includes monoclonal antibodies, immune checkpoint inhibitors, tumor vaccination, and adoptive T lymphocytes. These factors all show substantial efficacy for treating cancer in the clinic (Khalil et al., 2016). These immunotherapies awaken the human body's immune system to attack abnormal tumor cells with powerful cytokines, tumor vaccines, antibodies, and immune-stimulating adjuvants (Banstola et al., 2020). Compared with traditional methods of treating malignant tumors, especially chemoradiotherapy, immunotherapy is an innovative antitumor approach that dynamically regulates the immune system to assault cancer cells with multiple targets and directions (Tan et al., 2020).

The number of approved immunotherapy drugs has increased in recent years, and more combined treatment strategies, such as radiotherapy, chemotherapy or antitumor angiogenesis therapy, have been developed for the treatment of different types of cancer (Yu et al., 2019). However, a pivotal challenge we are facing in the widespread implementation of cancer immunotherapy is the controlled regulation of the immune system. On the one hand, these therapies have serious side effects, for instance, autoimmune and nonspecific inflammation are common (Riley et al., 2019); on the other hand, traditional immune stimulants lack the ability to target solid tumor tissue that results from many factors, including the tumor microenvironment (TME), immune evasion processes, and pharmacokinetics (Connor and Broome, 2018; Tan et al., 2020; Yang et al., 2021). In addition, because solid tumors face transmission barriers (such as complex TME), the main immunotherapy was initially evaluated in hematological malignancies. Given this factor, a fraction of immunotherapies, such as activated cytokines and immune checkpoint inhibitors (ICIs), have been licensed by the FDA as investments in the pharmaceutical market for solid tumors (Menon et al., 2016).

Even though many immunotherapies face challenges, surprisingly, the implementation of nanotechnology can effectively improve the efficiency of targeted delivery and the therapeutic efficacy of immune drugs (Li et al., 2020). Nanotechnology has become a trend in the field of medical science and has made great progress with the development of functional, engineered nanoparticles. Among them, gold nanoparticles (AuNPs) have been widely reported to guide an impressive resurgence and are highly remarkable (Hu et al., 2020). AuNPs can pass through the “EPR agent” (enhanced and retention effect) and specifically accumulate in tumor tissues and cells, which is highly beneficial for the targeted delivery of tumor vaccines and immune adjuvants (Riley et al., 2019). Recent studies have shown that nanoscience and technology continue to develop in the fields of tumor immunotherapy (Ou et al., 2020). AuNPs can not only be used for the targeted delivery of traditional antitumor immune adjuvants such as tumor-associated antigens and immune cytokines but also have become a research hotspot, such as in adoptive immune cell therapy and immune checkpoint inhibition therapy, which shows excellent potential clinical value (Savitsky and Yu, 2019). In this review, we summarized the application of AuNPs as immune agents, drug carriers or combinations with other immunotherapies for anticancer treatment.

## The Emergence of Nanotechnology for *Cancer* Immunotherapy

The conventional treatments for primary tumors are surgery, chemotherapy and radiotherapy. However, tumor recurrence and final treatment failure are still daunting challenges in the clinic. Indirect evidence from preclinical studies shows that the long-term success of cancer treatment lies in immunotherapy. Therefore, cancer immunotherapy is considered to be an effective treatment for the elimination of primary and metastatic tumors and the establishment of immune memory. Nanotechnology can simultaneously deliver various immunological reagents to the desired target site (tumor or lymph node). The ultimate application of nanomedicine is to reprogram or to regulate immune responses by accurately targeting biological pathways (Yu and De Geest, 2020). Hence, the emergence of nanotechnology provides a variety of materials and targeted properties to overcome many difficulties in immunotherapy. Nanoparticle systems have been widely used in the medical field and have many advantages compared with traditional methods (Hagan et al., 2018). Nanoparticles are used either as a protective delivery vehicle for a variety of cargo, improving the stability and solubility of their cargo, extending their half-life, or being used to target cancer cells (Qiu et al., 2017). There are many different applications of nanoparticle systems that can be used for immunotherapy in cancer. From the previously published literature, these methods include the delivery of vaccines and antibodies, and even more specifically, the targeting of specific cells such as antigen presenting cells (APCs) or dendritic cells and the modification of the tumor microenvironment to counteract many immunosuppressive effects of tumors (Qiu et al., 2017). It has been reported that polylactic-coglycolic acid (PLGA) nanoparticles (Surendran et al., 2018; Riley et al., 2019), liposomes, gold nanoparticles, and artificial exosomes are widely used for studies on the delivery of tumor immunotherapy drugs. First, this class of materials has the capacity to improve the synthesis process and to increase the modification of some molecules such as polyethylene glycol (PEG) and to develop its properties in biological distribution, pharmacokinetics, biological safety and other aspects. Second, these materials can not only utilize the modification of arginine-glycine-aspartic acid tripeptide (RGD) and other active targeting molecules to further improve its tumor targeting ability but can also deliver immune agonists to tumor tissues specifically and enhance the body’s antitumor immune response while reducing the probability of systemic inflammatory reactions. Therefore, nanotechnology is a candidate approach for enhancing cancer immunotherapy.

## The Application of AuNPs in *Cancer* Immunotherapy

### AuNPs as Nanocarriers for Immunotherapy

Gold nanoparticles (AuNPs) have attracted much attention due to their unique advantages among nanoparticles (Singh et al., 2018). In addition to their excellent targeting of tumor tissues and the immune system, AuNPs also have advantages compared with other metal nanoparticles. With the continuous development of nanotechnology, AuNPs are easily synthesizable in various shapes and sizes through chemical, physical or eco-friendly biological methods. AuNPs play many roles as multifunctional therapeutic agents, such as targeted delivery systems (vaccines, nucleic acids, and immune antibodies), theranostics and agents in photothermal therapy. They have also made great contributions in the field of biological imaging, such as radiotherapy, magnetic resonance angiography and photoacoustic imaging (Mioc et al., 2019).

First, gold nanoparticles are a kind of biologically inert material suitable for medical applications with strong plasticity (Boisselier and Astruc, 2009). Even delicate adjustment of the size and shape of AuNPs can lead to changes in the distribution, metabolism, cytotoxicity, immunogenicity and other properties of AuNPs. Moreover, AuNPs are highly modifiable, and the molecular density on their surface is higher than that of most other nanomaterials. AuNPs can conjugate molecules of different types and functions in a variety of ways while avoiding interference between these molecules. By simultaneously modifying molecules with different functions, such as PEG, RGD and immune adjuvants, researchers can improve many aspects of the performance of AuNPs and comprehensively enhance the efficacy of the targeted delivery of immune drugs and activation of the immune system (Lopez-Campos et al., 2019). More importantly, AuNPs can generate heat under a specific wavelength of laser irradiation due to their unique photodynamic properties. On the one hand, heat-related signaling stimulates immune factors in tumor tissues, inflammation, and transmitter secretion. On the other hand, it collaborats with the immune response for cancer cells and releases immune-activated drugs in cancer tissues, which achieves efficient and low toxicity of antitumor immune effects (Liu et al., 2018).

### The Nanocarrier Role of AuNPs for Drug Delivery

The application of nanotechnology is mainly based on the early detection and diagnosis of tumors by nanodevices that can selectively target chemotherapeutic drugs and deliver them to specific tumor sites. The special properties of AuNPs have long been regarded as potential tools for the diagnosis of various cancers and drug delivery applications. These properties include a high surface area to volume ratio, surface plasmon resonance, surface chemistry and multifunctionalization, facile synthesis, and a stable nature. Various types of drugs can be immobilized on the surface of AuNPs, most notably by direct -S or -N binding, ligand bonding, and adsorption by electrostatic interaction, van der Waals forces, and hydrogen bonding. In general, because of the stronger interaction between Au and S, -N binding holds more promise for delivering drugs in cancer cells than -S binding (Cheng et al., 2010). Moreover, the nontoxic and nonimmunogenic characteristics of AuNPs and their high permeability and retention provide additional benefits by enabling them to penetrate and to accumulate drugs easily at tumor sites (Lee and Choi, 2018). Various innovative approaches with AuNPs are under development. Of note, novel strategies, especially improved delivery strategies, can not only target tumors and immune cells more effectively but also increase the abundance of immunotherapeutics within lesions (Singh et al., 2018). Some materials, such as lipids, polymers, and metals, have been used to exploit delivery strategies (Lakshminarayanan et al., 2018). Currently, new delivery strategies for immunotherapy, including nanoparticles, scaffolds and hydrogels, are being researched and developed (Zhao et al., 2019). Among them, gold nanoparticles are particularly prominent.

#### Nanocarrier of Tumor Vaccines

**FIGURE 1:**
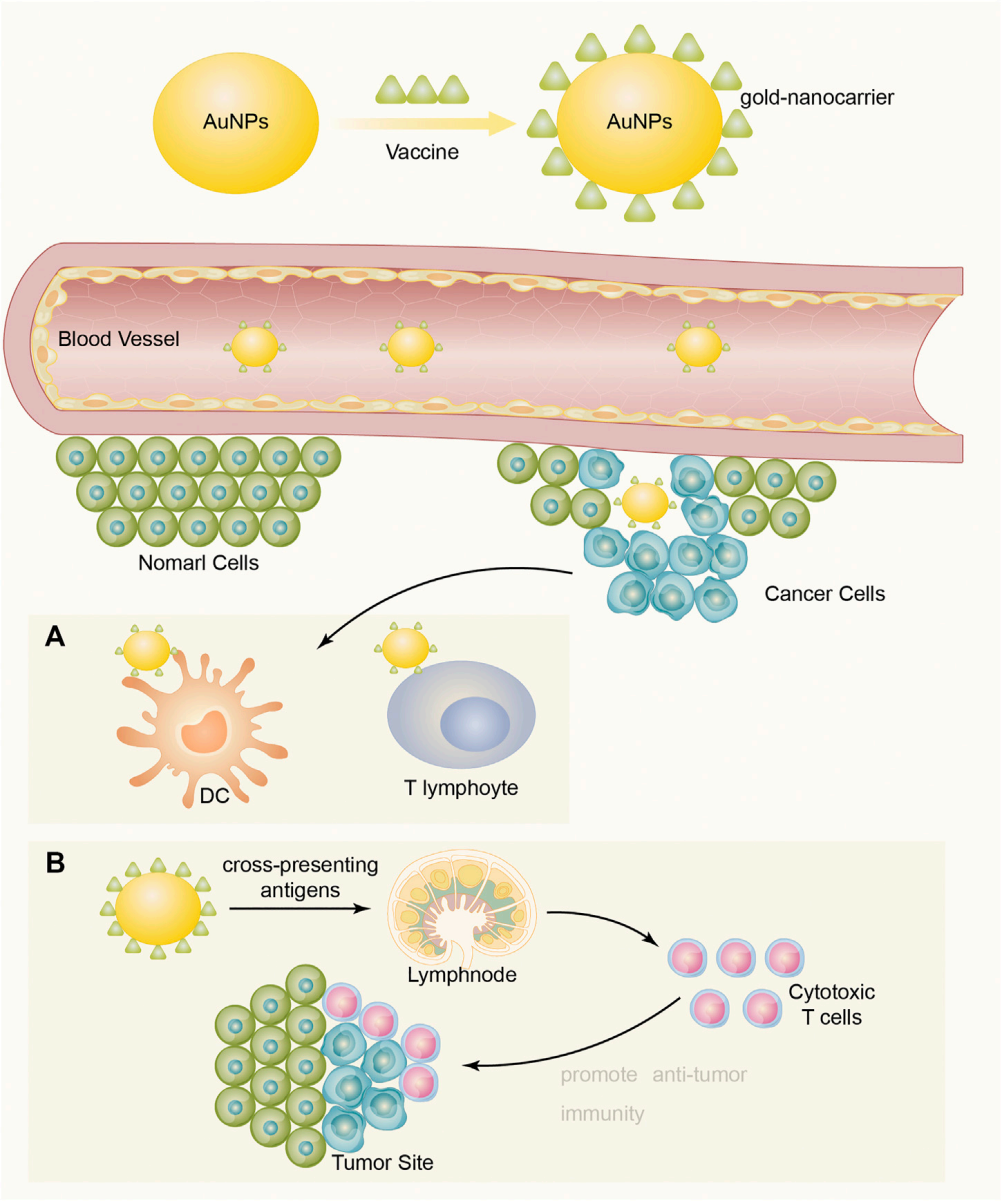
Vaccines connect to and display on the surface of AuNPs to become a formation of vaccine-AuNPs complex, then the complex penetrates blood vessels and delivers vaccine targeting cancer cells to enhance immune response. **(A)** Targeting for delivery to dendritic cells (DCs) or T lymphocytes. **(B)** Cross-present antigens to more effectively stimulate cytotoxic T lymphocytes and to promote antitumor immunity.

Tumor-associated antigen (TAA) reactivates the body's immune response to tumor cells and plays a vital role in the early prevention and treatment of cancer. In general, tumor vaccines may mainly consist of TAAs and adjuvants (Aly, 2012). Compared with direct injection of TAAs, AuNPs linked with vaccines are more suitable for protecting antigens from degradation and can be targeted for delivery to dendritic cells (DCs) or T lymphocytes. In addition, they are able to penetrate blood vessels and barriers and to be targeted to a specific cell by means of specifically functionalized molecules (Figure 1) AuNPs can also cross-present antigens to more effectively stimulate cytotoxic T lymphocytes and to promote antitumor immunity (Sehgal et al., 2014; Popescu and Grumezescu, 2015).

An excellent carrier is born at the right moment, and AuNPs have proven to be competent for the job because of the properties of AuNPs (such as the biocompatibility and nontoxicity of gold) (Zhou et al., 2016a). They show better performance than other nanoparticulate-based carriers; for example, their size is easily controlled for different applications, and the majority of antigens and adjuvants can be easily connected to and displayed on their surface. At the same time, AuNPs can be detected with noninvasive imaging techniques, providing clinicians with evidence on where vaccines have been delivered, which supports the evaluation of therapeutic efficacy (Dykman et al., 2018). A model of a hyaluronic acid (HA)- and antigen (ovalbumin, OVA)-decorated AuNP-based (HA-OVA-AuNP) vaccine (Cao et al., 2018) was invented for photothermally controlled cytosolic antigen delivery with near-infrared (NIR) irradiation and was discovered to induce antigen-specific CD8^+^ T-cell responses. HA-OVA-AuNPs promote antigen uptake by DCs through receptor-mediated endocytosis. HA-OVA-AuNPs show the ability to enhance NIR absorption and thermal energy translation. Cytosolic antigen delivery is allowed via the photothermally controlled process of partial heat-mediated endo/lysosome disruption by laser irradiation with reactive oxygen species generation, which helps to increase proteasome activity and downstream MHC I antigen presentation. Therefore, the HA-OVA-AuNP nanovaccine can effectively stimulate a potent anticancer immune response under laser irradiation. In another model of AuNPs mobilized with α-mannose as carriers for a TLR7 ligand to target immune cells (Shinchi et al., 2019). The small molecule synthetic TLR7 ligand 2-methoxyethoxy-8-oxo-9-(4-carboxy benzyl) adenine (1V209) and α-mannose were coimmobilized via linker molecules consisting of thioctic acid on the AuNP surface (1V209-αMan-AuNPs). Compared with the unconjugated 1V209 derivative in mouse bone marrow-derived dendritic cells and in human peripheral blood mononuclear cells, the 1V209-αMan-AuNPs showed higher extracorporeal cytokine production activity. In the internal immunization study, 1V209-αMan-AuNPs induced obviously higher titers of IgG2c antibody specific to ovalbumin as an antigen than unconjugated 1V209, and splenomegaly and weight loss were not apparent. These results suggest that 1V209-αMan-AuNPs could be safe and effective adjuvants for the development of vaccines against cancer.

Kang et al. (Kang et al., 2018) designed a nanodelivery system that delivers TAA to natural killer cells and APCs. The calcium phosphate nucleus of the delivery system was coated with melanoma TAA, αHSP70P protein and adjuvant CpG, and then, the calcium nucleus was coated with a phospholipid bilayer. Effective lymphocyte metastasis and multiepitope T lymphocyte responses were observed when the nanoparticles were injected into mice *in vivo*. This approach can also induce the expansion of CD8^+^ T lymphocytes and NKG2D + NK cell subsets. The nanoparticles also had synergistic effects on the maturation and antigen presentation of DCs derived from bone marrow.

AuNPs, as carriers of tumor vaccines, play an important role in antitumor immunotherapy. Taken together, AuNP‐based vaccines are novel and efficient antitumor treatments (Ahn et al., 2014).

#### Nanocarrier Delivery of Genetic Drugs

The delivery of small interfering RNA (siRNA) mediated by nanocarriers has provided a novel method of intracellular antigen synthesis, which has great application value in antitumor immunotherapy (Yin et al., 2014). Under normal physiological conditions, the inherent negative charge of siRNAs is usually degraded by related enzymes, and siRNA is difficult to be transferred to the target location (Liu et al., 2019a). There are two main reasons why gold nanoparticles (AuNPs) can be used as effective nanomaterials for siRNA delivery applications (Gindy and Prud'homme, 2009; Mahmoodi Chalbatani et al., 2019). First, functional diversity can be easily obtained with the creation of multifunctional monolayers. Second, because of the low toxicity, low size disparity and selective gene silencing and transfection. So far, AuNPs are one of the most extensively used carrier tool for siRNA as an anti-cancer strategy.

In the research of Hou et al. (2016), the authors designed nonviral pDNA/siRNA delivery vectors, that is, generation 5 dendrimer-entrapped gold nanoparticles (Au DENPs) partially modified with polyethylene glycol monomethyl ether. The entrapped Au DENPs were effectively used to deliver Bcl-2 (B-cell lymphoma 2) siRNA to human cervical cancer cell lines to silence the enhanced green fluorescent protein and luciferase reporter genes. Suman’s article reported a model used to treat melanoma comprising layer-by-layer assembled gold nanoparticles (LbL-AuNPs) containing anti-STAT3 siRNA and IM (imatinib mesylate) (Labala et al., 2017). Compared with LbL-AuNPs containing either STAT3 siRNA or IM, the treatment showed greater inhibition of STAT3 protein, reduced cell viability and increased apoptotic events. In summary, combining AuNPs with the RNAi pathway by delivering siRNA and small molecule drugs (IMs) is a way of creating a drug delivery system.

Only if when siRNA was sent to the cytoplasm where gene silencing takes place, it could be of value (Elbashir et al., 2001). Previous studies have shown that functional Au DENPs can transport siPDL1 (programmed siRNA-PD-L1) to cancer cells, effectively down-regulate the expression of PD-L1 protein, and increase the infiltration of CD8^+^ and CD4^+^ T cells in tumor tissues and spleen, thereby promoting immunotherapy. Its tumor suppression efficiency is much higher than that of PD-L1 antibody (Xue et al., 2021). So siRNA-AuNPs delivery system may have great application potential in immunotherapy.

#### AuNPs Delivery of Immune Antibodies

Currently, approved therapies for PD-1/PD-L1 have been effectively used to improve the survival and quality of life of cancer patients with chemotherapy and targeted drug tolerance by using nivolumab, pembrolizumab, cemiplimab, atezolizumab, durvalumab, and avelumab to effectively inhibit the binding of PD-1 to PD-L1 and to prevent the immune escape of cancer cells through the use of antibody drugs, such as nivolumab, pembrolizumab, cemiplimab, atezolizumab, durvalumab, and avelumab (Wang et al., 2019). Despite their numerous advantages, these antibody drugs still have many disadvantages, such as high cost of use, low clinical response rate (approximately 20%), influence of individual differences, large required therapeutic dose, high probability of causing immune side effects, and cases of developing resistance (Luo et al., 2018). To overcome some of these shortcomings, many studies are focusing on immunotherapy strategies that combine immune antibodies with AuNPs.

In fact, the most important antitumor molecules used in clinical practice are PD-1, CTLA-4, Tim-3 and LAG-3, which are the main immune checkpoint molecules associated with tumors (Qin et al., 2019). One extremely promising approach to achieve anticancer immunity is to block the immune checkpoint pathway mechanism of cancer cells and camouflaging the conventional components of the human body (Li et al., 2019). These molecules are expressed in immune cells and can interact with corresponding ligands expressed in cancer cells or immunomodulatory cells in the tumor microenvironment to inhibit the cellular immune response and cause immune escape of cancer cells. Immune checkpoint inhibitors are another kind of mainstream immune anticancer method that has entered the clinic (Darvin et al., 2018). This class of drugs can block the interaction between immune checkpoints and their ligands and restore immune cells to recognize and to kill cancer cells (Han et al., 2020).

**FIGURE 2:**
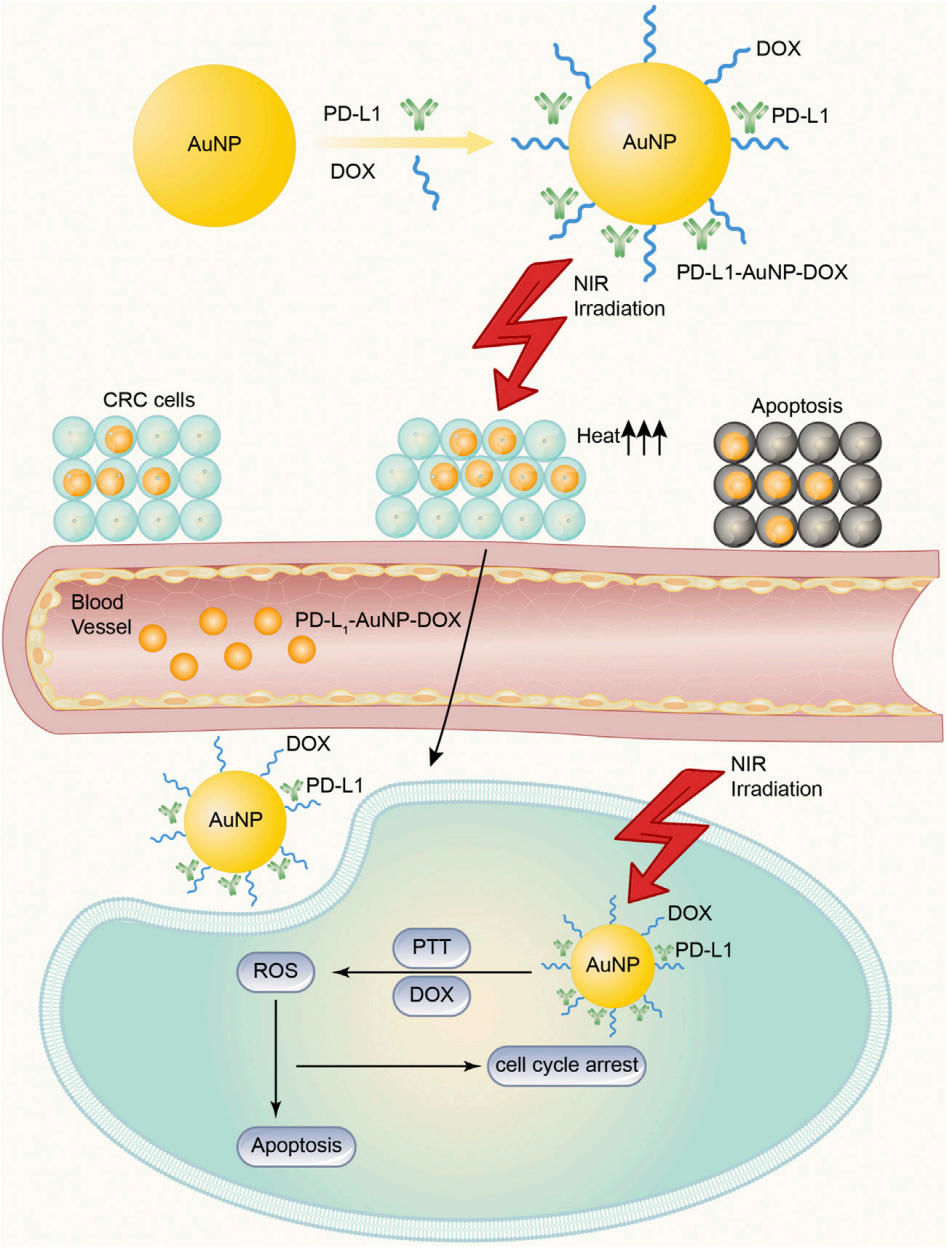
DOX-conjugated and anti-PD-L1 targeting gold nanoparticles (PD-L1-AuNPs-DOX) model for CRC. The PD-L1-AuNPs-DOX model, under the NIR irradiation, may improve the ability of targeted delivery, and gather PD-L1 and DOX together in cancer cells more easily. Then the model effectively generates ROS to increase apoptosis and cell cycle arrest to suppress the *in vitro* proliferation of CRC cells.

PD-1 has received much attention as an immune checkpoint in clinical anticancer therapy (Gong et al., 2018). It is usually expressed in T cells and interacts with the PD-L1 receptor overexpressed on the surface of cancer cells or immunosuppressive cells to inhibit the immune response of T cells to tumor cells and to induce the apoptosis of T cells (Liu et al., 2021; Xia et al., 2019). To overcome some of these shortcomings, Emami et al. (2019) designed doxorubicin (DOX)-conjugated and anti-PD-L1 targeting gold nanoparticles (PD-L1-AuNPs-DOX) for colorectal cancer (CRC). Despite drug resistance, DOX and PD-L1 antibodies are difficult to deliver to tumor sites because of the barrier of the tumor microenvironment (TME) and other factors. Therefore, the authors constructed a model that may improve the drug delivery ability. First, AuNPs exhibit characteristic surface plasma resonance (SRS) absorption in the near-infrared (NIR) region (Banstola et al., 2018), and AuNP-based photothermal therapy (PTT) can be used to ablate tumors by turning NIR light energy into heat and generating of reactive oxygen species (ROS). At the same time, DOX can also be loaded onto AuNP platforms, which enables DOX and heat to be delivered specifically and simultaneously to tumor microenvironments (Chen et al., 2017). Second, some CRC subtypes, especially microsatellite instability-high (MSI-H) CRC (a highly immunogenic cancer), show PD-L1 upregulation on cell surfaces and PD-L1 overexpression, which is known to be distinct for prognosis and survival in CRC patients. Therefore, the authors aimed to construct AuNPs modified with an anti-PD-L1 antibody and drug-covalent conjugation to lipoic acid polyethylene glycol N-hydroxy succinimide (LA-PEGNHS) as a novel drug delivery system for the combined delivery of a drug and heat to CRC cells. In brief, the PD-L1-AuNPs-DOX model (Figure 2) successfully facilitated the efficient intracellular uptake of DOX and NIR irradiation obviously and synergistically suppressed the *in vitro* proliferation of CRC cells by increasing apoptosis and cell cycle arrest, and this model in combination with synergistic targeted chemo-photothermal therapy has potential for the treatment of localized CRC. In addition, using AuNPs to deliver PD-1/PD-L1 antibodies or siRNA is another effective way to inhibit PD-1 tumor immune checkpoints. Meir's team (Meir et al., 2017) modified the PD-L1 antibody to adhere to the surface of AuNPs and effectively improved the concentration of antibody drugs at tumor sites by using the efficient targeted drug release ability of AuNPs. Liu et al. (2019b) loaded PD-L1 siRNA into gold nanocarriers to knock down the expression of PD-L1 in tumor cells, which also achieved a good tumor inhibition effect. At present, 5 more research groups have attempted to use AuNPs to deliver PD-1/PD-L1 inhibitors and other anticancer drugs to explore drug combination strategies based on PD-1/PD-L1 targets. The results showed that the combination of PD-L1 antibody mediated by AuNPs and doxorubicin could not only enhance the induction of apoptosis of cancer cells but also inhibit tumor stem cell-mediated angiogenesis by inhibiting autophagy of cancer cells and thus inhibit tumor recurrence (Emami et al., 2019; Ruan et al., 2019).

## Application Strategies of AuNPs to Improve the Tumor Microenvironment

### The Role of AuNPs in the Tumor Microenvironment

Due to the influence of tumor growth, the TME possesses premium physiological characteristics, including hypoxia, slight acidity, and vascular irregularity. In addition, the TME can generate an immunosuppressive microenvironment by releasing cytokine mediators and gathering immunosuppressive cells (Dunn et al., 2004; Estrella et al., 2013). The study by Ibrahim et al. (2018) signified that AuNPs with a size of 5, 20 and 50 nm can up-regulate interleukin (IL)-1β, IL-6, and tumor necrosis factor in the liver, spleen, and kidney of mice at a certain dose. TNF-α and other immune cytokines expression levels. Then the hypoxic state was improved because of the variation of these cytokine level. At the same time, both immune cell infiltration and the efficacy of tumor immunotherapy were enhanced at tumor sites.

Moreover, the special pathological structure of tumor and its inhibitory immune microenvironment jointly limit the efficacy of immunotherapy. Many advances have been made in reshaping the pathological structure of tumor that affects the efficacy of immunotherapy and enhancing the efficacy of immunotherapy. Consequently, remodeling the tumor immunosuppressive microenvironment is of great significance for tumor immunotherapy (Hou et al., 2020). Insufficient tumor vascular perfusion can lead to highly hypoxic TME, which is closely related to immunosuppressive TME. Regulating the normalization of blood vessels at tumor sites is of great significance for remodeling the immunosuppressive tumor microenvironment and enhancing immune cell infiltration in tumor sites (Mazzieri et al., 2011). AuNPs can improve vascular morphology, increase vascular perfusion, reduce tumor hypoxia, and inhibit the migration of HUVECs and tumor cells (Li et al., 2017). In the research of Li et al. (2017), they connoted that AuNPs can regulate the angiogenin-angiogenin type I receptor pathway, promote the normalization of tumor blood vessels, increase blood perfusion and alleviate hypoxia at the tumor site.

In additional, immune response requires the participation of immune cells, including dendritic cells (DCs) and macrophages, and they mediate innate immune response, which can play the pro- and anti-tumor effect depending on the inflammatory mediators and cytokines in TME (Yang et al., 2021). The growth and development of tumor cells leads to a hypoxic situation, and this state subsequently modifies the physiological function of microenvironment. To this end, anti-tumor drugs can hardly penetrate TME and target tumor cells (Rajendrakumar et al., 2018). With the deepening of the research on AuNPs, how AuNPs work in tumor immunotherapy for TME have been report. Firstly, in comparison with the regular immunotherapy drugs, AuNPs could modulate the immunosuppressive environment in TME through targeting various of abnormal components of TME. For example, the abnormal secretion of vascular endothelial growth factor (VEGF) and transforming growth factor β (TGF-β) inhibit the immune response of DCs, and transfer macrophages to the pro-tumorigenic M2 phenotype. Specifically designed AuNPs combined immunotherapy drugs can target these abnormal components in TME and transform the immunosuppressive TME to an immunosupportive state, thereby improving the efficacy of cancer immunotherapy (Overchuk and Zheng, 2018).

However, what worth noting is that AuNPs alone may be unlikely to directly impact the immune response, because their effects on the immune system remain ill-defined. Dey et al. (2021) created an *in vitro* cell model. They used primary macrophages and DCs of mice as an APC model. Even though AuNPs indistinctively changed the functions of macrophages and DCs, AuNPs had different effects on the response of macrophages and DCs to subsequent stimulation. Firstly, the secretion level of cytokines and chemokine were altered in DCs and macrophages. Moreover, antigen presentation to T cells increased when DCs came into contact with AuNPs resulting in stronger Th1, Th2 and Th17 responses. That is why we emphasize that immunotherapy plus AuNPs do not directly alter the immune response but indirectly influence the function of DCs and macrophages by modulating abnormal components in TME to get twice the result with half the effort. Therefore, AuNPs have been widely designed as drug delivery system.

### Anti-Angiogenesis and Vascular Normalization Strategies of AuNPs

Abnormal appearance and impaired function are the most common features of tumor vasculature (Krishna Priya et al., 2016). Hypoxia in the TME induces continued production of proangiogenic molecules, such as vascular endothelial growth factor (VEGF) and transforming growth factor-β (TGF-β) (De Palma et al., 2017; Maity et al., 2000; Hinz, 2015). The imbalance between proangiogenic and antiangiogenic factors leads to rapid but abnormal tumor angiogenesis. Owing to the detachment of the pericytes surrounding the endothelial cells, the blood vessels have increased permeability (Barlow et al., 2013; Huang et al., 2018). Hence, aberrant vasculature contributes greatly to abnormalities in the TME.

If abnormal tumor vasculature is the key issue that hinders the implementation of tumor treatment, then vascular normalization strategies could be a promising solution. Another problem coming to us subsequently is that the therapeutic efficacy of antiangiogenic therapy has been obstructed by acquired resistance of the endothelium toward antiangiogenic drugs such as anti-VEGF therapy (Mattheolabakis and Mikelis, 2019). Zhang et al. (2019a) investigated the use of AuNPs as a therapeutic tool to disrupt multicellular crosstalk in TME cells, with a focus on inhibiting angiogenesis. The authors showed that CM (conditioned media) from ovarian CCs, CAFs, or ECs themselves induced tube formation and migration of ECs *in vitro*. These results prove that AuNPs inhibit angiogenesis by blocking VEGF-VEGFR2 signaling from TME cells to endothelial cells.

Although the antiangiogenic mechanism of AuNPs is still unclear, Pan et al. (2013) revealed that it may be due to the effect of AuNPs on VEGF signaling; AuNPs can reduce VEGF165-induced VEGFR2 and AKT phosphorylation. However, a single vascular normalization strategy cannot improve its penetration in tumor tissues, and there is still a need to develop new formulations based on AuNPs combined with other therapies (Chauhan et al., 2012), such as immunotherapy.

Successful immunotherapy requires not only the infiltration of immune cells, but also an immune-supporting microenvironment to maintain the proliferation and function of  T cells. Normalization of blood vessels provides such an environment for immunotherapy (Huang et al., 2013; Jain, 2013). It is also important to note that dysfunctional vessels restrict the infiltration of immune cells as well as the efficient delivery of nanoparticles and therapeutic drugs (Azzi et al., 2013). Meanwhile, some reports have highlighted the effect of mediating tumor vascular normalization of AuNPs. Huang et al. (2020) focused on a model, named targeting polymer and folic acid-modified gold nanoparticles (AuNPP-FA), which can both restrain tumor angiogenesis and promote vascular normalization. It is because of the increased infiltration of CD3^+^CD8^+^ T lymphocytes that the immunotherapeutic response was enhanced by decreasing vascular permeability and improving vascular perfusion. Thereby, the vascular normalization strategy of AuNPs has great potential for tumor immunotherapy.

## Photothermal Therapy and Photodynamic Therapy of AuNPs

AuNPs, with their multiple unique functional properties and ease of synthesis, have attracted extensive attention in antitumor tactics. Their inherent features can be altered by changing the characterization of the nanoparticles, such as shape, size and aspect ratio. They can be applied to a wide range of medical applications, especially photothermal therapy (PTT) and photodynamic therapy (PDT) (Hu et al., 2020). AuNPs exhibit favorable physical properties and tailored surface functionalization, providing a potential platform for developing cancer immunotherapy (Guo et al., 2017).

### Application of Photothermal Therapy

The reason why PTT is available under the stimulation of pulsed or continuous-wave Doppler (CW) visible lasers is the surface plasmon resonance (SPR) absorption of AuNPs in the visible region, whereby such treatment may be suitable for shallow tumors (i.e., skin cancer) (El-Sayed et al., 2006; Huang et al., 2007). Despite being a standalone therapy, gold nanoparticle-mediated PTT has recently been developed in combination with other therapies, such as chemotherapy, gene drugs, and immunotherapy, for enhanced antitumor effects (Riley and Day, 2017). Here, we emphatically introduce the combination of PTT and immunotherapy, which is based on the phenomenon that heat causes dying cancer cells to release antigens and heat shock proteins (HSPs) that are captured by antigen-presenting cells such as DCs to mediate an immune response (Almeida et al., 2014; Zhou et al., 2016b). Moreover, the immune environment created by PTT can strengthen immunotherapies to prolong anticancer effects. Liu et al. (2019c) constructed a new nanoplatform GNS@CaCO3/Ce6-NK by loading CaCO3-coated gold nanostars (GNSs) with chlorin e6 molecules (Ce6) into human peripheral blood mononuclear cell (PBMC)-derived NK cells for tumor-targeted therapy. This approach was used because the authors hypothesized that Ce6 would remain stable with characteristic NIR absorption during the endocytic process of NK cells and that the new platform fully utilized the immune function of NK cells and the physical properties of AuNPs to prove the synergistic therapeutic effects of PTT and immunotherapy. Combining PTT with NK cells increased the efficiency and accuracy of the tumor-targeting ability compared to other immunotherapies alone. Thus, this platform reflects a prominent synergistic strategy for enhanced PTT and immunotherapy in the area of anticancer development.

### Application of Photodynamic Therapy

PDT is another light therapy developed in recent decades to destroy cancer cells and pathogenic bacteria (Abrahamse and Hamblin, 2016). PDT involves visible light, a photosensitizer (PS), and molecular oxygen (O_2_) from tissues. If PDT desires to function successfully, it is completely dependent on the availability of O_2_ in tissues. In the process of PDT, the PS absorbed by the tissue is excited by laser light of a specific wavelength. Irradiating the tumor site can activate PS, which selectively accumulates in the tumor tissue, triggering a photochemical reaction to destroy the tumor. The excited PS will transfer energy to the surrounding O_2_ to generate reactive oxygen species (ROS) and to increase ROS levels in the target sites. ROS can react with adjacent biological macromolecules to produce significant cytotoxicity, cell damage, and even death or apoptosis (Imanparast et al., 2018; Falahati et al., 2020). Liang et al. (2018) reported a gold nanoparticle system based on core-shell gold nanocage@manganese dioxide (AuNC@MnO2, AM) nanoparticles as tumor microenvironment responsive oxygen producers and NIR-triggered reactive ROS generators for oxygen-boosted immunogenic PDT against metastatic triple-negative breast cancer (mTNBC). In this model, the MnO_2_ shell degrades in an acidic TME and generates massive oxygen to boost the PDT effect of AM nanoparticles under laser irradiation and to ameliorate local hypoxia. Furthermore, in the oxygen-boosted PDT effect, immunogenic cell death (ICD) is elicited by damage-associated molecular pattern (DAMP) release, which induces DC maturation and effector cell activation, thereby strongly evoking systematic antitumor immune responses against mTNBC. Hence, this nanosystem offers a promising approach to ablate primary tumors while preventing tumor metastasis through immunogenic endoscopic effects.

### Other Application Strategies

In addition to PTT and PDT, radiation therapy (RT) is one of the least invasive and commonly used methods in the treatment of various cancers (Sztandera et al., 2019). RT involves the delivery of high-intensity ionizing radiation (such as γ-rays and X-rays) to tumor tissues while simultaneously protecting the surrounding healthy cells, tissues, and organs, resulting in the death of tumor cells (Retif et al., 2015; Klębowski et al., 2018). Recently, there have been many reports of radiosensitization using AuNPs in RT, which is due to the high atomic number of gold nanoparticles (Jain et al., 2011; McMahon et al., 2011). The most likely mechanism of radiosensitization from AuNPs is that Auger electron production from the surface of the AuNPs can increase the production of ROS, reduce the total dose of radiation, and increase the dose administered locally to the tumor sites, eventually resulting in cell death. These methods also provide potential ways to expose tumor antigens and to enhance the response to cancer immunotherapy (Jeynes et al., 2014; Retif et al., 2015).

## Clinical Application of AuNPs

The use of nanoparticles in therapeutic applications has been improved by coating gold with organic compounds, such as amino acids and amino sugars, which act as carriers to transport nanoparticles to tumor cells (Daniel and Astruc, 2004; Tshikhudo et al., 2004). Colloidal gold-based nanoparticles have been designed to target the delivery of tumor necrosis factor (TNF) and paclitaxel to solid tumors, introducing AuNPs as tumor-targeted drug delivery vectors (Paciotti et al., 2006). AuNPs are recognized as excellent drug and anticancer carriers with many biochemical and therapeutic applications (Singh et al., 2018). In a clinical trial approved by the FDA, whose first phase has been completed, novel PEGylated AuNPs were utilized to deliver TNF into cancer cells, ending with selective TNF storage in tumor cells (Libutti et al., 2010).

However, very few clinical trials are being actively carried out for the approval of AuNPs for cancer diagnostics and therapy (Tomić et al., 2014; Qiu et al., 2015). As described above, AuNPs show potential for use in cancer diagnostics and therapeutics. However, the absence of coherent information on the actual effect of nanoparticles could have deleterious effects and a negative impact on human health. For example, the toxicity and chain reaction when AuNPs are used *in vivo* are not abundantly clear because few teams have tested them in clinical trials. Thus, there is controversy and disagreement about the potential of AuNPs for clinical use at present (Cheng et al., 2012).

## Perspective

The advantages of AuNPs make them more effective in the immunotherapy of malignant tumors. For example, the ability to accumulate at tumor sites and the photothermal properties can enable the efficient and targeted delivery of genes, cancer vaccines, immune antibodies and other immune therapeutic-related components. Some studies have shown that in combination with current popular fields of tumor immunotherapy, such as PD-1/PD-1 AuNPs demonstrate excellent efficacy and show great potential clinical application value (Pietro et al., 2016; Liu et al., 2019b). It has been demonstrated that the targeted delivery of immunoregulatory molecules by AuNPs can not only eliminate killed primary tumor tissue but also promote a systemic immune response to treat metastatic lesions and prevent tumor recurrence (Zhang et al., 2019b).

However, AuNPs have various disadvantages in their application (Singh et al., 2018). First, AuNPs cannot be degraded and easily accumulate *in vivo* during long-term treatment, causing uncertain side effects. Second, the application of the photothermal effect of AuNPs is often limited by the penetration depth of near-infrared lasers, and the heating intensity will decrease with increasing laser penetration depth, which limits the directional drug release effect and immunoregulatory activity of AuNPs. More importantly, a series of pharmacokinetic and histocompatibility parameters of AuNPs are changed by surface modification (Weintraub, 2013).

## Conclusion

Therefore, AuNPs are candidates for enhancing the efficiency and safety of cancer immunotherapy. Every modification of the surface radical groups and ligands of AuNPs requires a reevaluation of their pharmacological and toxicological effects, which increases the amount and cycle of related drug research and development work. Nevertheless, the shortcomings of AuNPs have not deterred researchers from using them to improve the efficacy of tumor immunotherapy. In the future, there will be a series of works to modify the characteristics of AuNPs to overcome these shortcomings.

## References

[B1] Abrahamse H. Hamblin M. R. (2016). New Photosensitizers for Photodynamic Therapy. Biochem. J. 473, 347–364. 10.1042/bj20150942 26862179 PMC4811612

[B2] Ahn S. Lee I. H. Kang S. Kim D. Choi M. Saw P. E. (2014). Gold Nanoparticles Displaying Tumor-Associated Self-Antigens as a Potential Vaccine for Cancer Immunotherapy. Adv. Healthc. Mater. 3, 1194–1199. 10.1002/adhm.201300597 24652754

[B3] Almeida J. P. Figueroa E. R. Drezek R. A. (2014). Gold Nanoparticle Mediated Cancer Immunotherapy. Nanomedicine 10, 503–514. 10.1016/j.nano.2013.09.011 24103304 PMC3966952

[B4] Aly H. A. (2012). Cancer Therapy and Vaccination. J. Immunol. Methods 382, 1–23. 10.1016/j.jim.2012.05.014 22658969

[B5] Azzi S. Hebda J. K. Gavard J. (2013). Vascular Permeability and Drug Delivery in Cancers. Front. Oncol. 3, 211. 10.3389/fonc.2013.00211 23967403 PMC3744053

[B6] Banstola A. Jeong J. H. Yook S. (2020). Immunoadjuvants for Cancer Immunotherapy: A Review of Recent Developments. Acta Biomater. 114, 16–30. 10.1016/j.actbio.2020.07.063 32777293

[B7] Banstola A. Emami F. Jeong J.-H. Yook S. (2018). Current Applications of Gold Nanoparticles for Medical Imaging and as Treatment Agents for Managing Pancreatic Cancer. Macromol. Res. 26, 955–964. 10.1007/s13233-018-6139-4

[B8] Barlow K. D. Sanders A. M. Soker S. Ergun S. Metheny-Barlow L. J. (2013). Pericytes on the Tumor Vasculature: Jekyll or Hyde? Cancer Microenviron 6, 1–17. 10.1007/s12307-012-0102-2 22467426 PMC3601214

[B9] Boisselier E. Astruc D. (2009). Gold Nanoparticles in Nanomedicine: Preparations, Imaging, Diagnostics, Therapies and Toxicity. Chem. Soc. Rev. 38, 1759–1782. 10.1039/b806051g 19587967

[B10] Cao F. Yan M. Liu Y. Liu L. Ma G. (2018). Photothermally Controlled MHC Class I Restricted CD8+ T-Cell Responses Elicited by Hyaluronic Acid Decorated Gold Nanoparticles as a Vaccine for Cancer Immunotherapy. Adv. Healthc. Mater. 7, e1701439. 10.1002/adhm.201701439 29508543

[B11] Chauhan V. P. Stylianopoulos T. Martin J. D. Popović Z. Chen O. Kamoun W. S. (2012). Normalization of Tumour Blood Vessels Improves the Delivery of Nanomedicines in a Size-dependent Manner. Nat. Nanotechnol 7, 383–388. 10.1038/nnano.2012.45 22484912 PMC3370066

[B12] Chen Y. Li H. Deng Y. Sun H. Ke X. Ci T. (2017). Near-infrared Light Triggered Drug Delivery System for Higher Efficacy of Combined Chemo-Photothermal Treatment. Acta Biomater. 51, 374–392. 10.1016/j.actbio.2016.12.004 28088668

[B13] Cheng Y. Samia A. C. Li J. Kenney M. E. Resnick A. Burda C. (2010). Delivery and Efficacy of a Cancer Drug as a Function of the Bond to the Gold Nanoparticle Surface. Langmuir 26, 2248–2255. 10.1021/la902390d 19719162

[B14] Cheng Z. Al Zaki A. Hui J. Z. Muzykantov V. R. Tsourkas A. (2012). Multifunctional Nanoparticles: Cost versus Benefit of Adding Targeting and Imaging Capabilities. Science 338, 903–910. 10.1126/science.1226338 23161990 PMC3660151

[B15] Connor D. M. Broome A. M. (2018). Gold Nanoparticles for the Delivery of Cancer Therapeutics. Adv. Cancer Res. 139, 163–184. 10.1016/bs.acr.2018.05.001 29941104

[B16] Daniel M. C. Astruc D. (2004). Gold Nanoparticles: Assembly, Supramolecular Chemistry, Quantum-Size-Related Properties, and Applications toward Biology, Catalysis, and Nanotechnology. Chem. Rev. 104, 293–346. 10.1021/cr030698+ 14719978

[B17] Darvin P. Toor S. M. Sasidharan Nair V. Elkord E. (2018). Immune Checkpoint Inhibitors: Recent Progress and Potential Biomarkers. Exp. Mol. Med. 50, 1–11. 10.1038/s12276-018-0191-1 PMC6292890 30546008

[B18] De Palma M. Biziato D. Petrova T. V. (2017). Microenvironmental Regulation of Tumour Angiogenesis. Nat. Rev. Cancer 17, 457–474. 10.1038/nrc.2017.51 28706266

[B19] Dey A. K. Gonon A. Pécheur E.-I. Pezet M. Villiers C. Marche P. N. (2021). Impact of Gold Nanoparticles on the Functions of Macrophages and Dendritic Cells. Cells 10 (1), 96. PMID: 33430453; PMCID: PMC7826823. 10.3390/cells10010096 PMC7826823 33430453

[B20] Dunn G. P. Old L. J. Schreiber R. D. (2004). The Immunobiology of Cancer Immunosurveillance and Immunoediting. Immunity 21, 137–148. 10.1016/j.immuni.2004.07.017 15308095

[B21] Dykman L. A. Staroverov S. A. Fomin A. S. Khanadeev V. A. Khlebtsov B. N. Bogatyrev V. A. (2018). Gold Nanoparticles as an Adjuvant: Influence of Size, Shape, and Technique of Combination with CpG on Antibody Production. Int. Immunopharmacol 54, 163–168. 10.1016/j.intimp.2017.11.008 29149704

[B22] El-Sayed I. H. Huang X. El-Sayed M. A. (2006). Selective Laser Photo-thermal Therapy of Epithelial Carcinoma Using Anti-EGFR Antibody Conjugated Gold Nanoparticles. Cancer Lett. 239, 129–135. 10.1016/j.canlet.2005.07.035 16198049

[B23] Elbashir S. M. Harborth J. Lendeckel W. Yalcin A. Weber K. Tuschl T. (2001). Duplexes of 21-nucleotide RNAs Mediate RNA Interference in Cultured Mammalian Cells. Nature 411 (6836), 494–498. PMID: 11373684. 10.1038/35078107 11373684

[B24] Emami F. Banstola A. Vatanara A. Lee S. Kim J. O. Jeong J. H. (2019). Doxorubicin and Anti-PD-L1 Antibody Conjugated Gold Nanoparticles for Colorectal Cancer Photochemotherapy. Mol. Pharm. 16, 1184–1199. 10.1021/acs.molpharmaceut.8b01157 30698975

[B25] Estrella V. Chen T. Lloyd M. Wojtkowiak J. Cornnell H. H. Ibrahim-Hashim A. (2013). Acidity Generated by the Tumor Microenvironment Drives Local Invasion. Cancer Res. 73, 1524–1535. 10.1158/0008-5472.Can-12-2796 23288510 PMC3594450

[B26] Falahati M. Attar F. Sharifi M. Saboury A. A. Salihi A. Aziz F. M. (2020). Gold Nanomaterials as Key Suppliers in Biological and Chemical Sensing, Catalysis, and Medicine. Biochim. Biophys. Acta Gen. Subj 1864, 129435. 10.1016/j.bbagen.2019.129435 31526869

[B27] Gindy M. E. Prud'homme R. K. (2009). Multifunctional Nanoparticles for Imaging, Delivery and Targeting in Cancer Therapy. Expert Opin. Drug Deliv. 6, 865–878. 10.1517/17425240902932908 19637974

[B28] Gong J. Chehrazi-Raffle A. Reddi S. Salgia R. (2018). Development of PD-1 and PD-L1 Inhibitors as a Form of Cancer Immunotherapy: A Comprehensive Review of Registration Trials and Future Considerations. J. Immunother. Cancer 6, 8. 10.1186/s40425-018-0316-z 29357948 PMC5778665

[B29] Guo J. Rahme K. He Y. Li L. L. Holmes J. D. O'Driscoll C. M. (2017). Gold Nanoparticles Enlighten the Future of Cancer Theranostics. Int. J. Nanomedicine 12, 6131–6152. 10.2147/IJN.S140772 28883725 PMC5574664

[B30] Hagan C. T. Medik Y. B. Wang A. Z. (2018). Nanotechnology Approaches to Improving Cancer Immunotherapy. Adv. Cancer Res. 139, 35–56. 10.1016/bs.acr.2018.05.003 29941106 PMC11811840

[B31] Han Y. Liu D. Li L. (2020). PD-1/PD-L1 Pathway: Current Researches in Cancer. Am. J. Cancer Res. 10, 727–742. 32266087 PMC7136921

[B32] Hegde P. S. Chen D. S. (2020). Top 10 Challenges in Cancer Immunotherapy. Immunity 52, 17–35. 10.1016/j.immuni.2019.12.011 31940268

[B33] Hinz B. (2015). The Extracellular Matrix and Transforming Growth Factor-Β1: Tale of a Strained Relationship. Matrix Biol. 47, 54–65. 10.1016/j.matbio.2015.05.006 25960420

[B34] Hou P. Kapoor A. Zhang Q. Li J. Wu C.-J. Li J. (2020). Tumor Microenvironment Remodeling Enables Bypass of Oncogenic KRAS Dependency in Pancreatic Cancer. Cancer Discov. 10, 1058–1077. 10.1158/2159-8290.Cd-19-0597 32341020 PMC7334087

[B35] Hou W. Wei P. Kong L. Guo R. Wang S. Shi X. (2016). Partially PEGylated Dendrimer-Entrapped Gold Nanoparticles: A Promising Nanoplatform for Highly Efficient DNA and siRNA Delivery. J. Mater. Chem. B 4, 2933–2943. 10.1039/c6tb00710d 32262971

[B36] Hu X. Zhang Y. Ding T. Liu J. Zhao H. (2020). Multifunctional Gold Nanoparticles: A Novel Nanomaterial for Various Medical Applications and Biological Activities. Front. Bioeng. Biotechnol. 8, 990. 10.3389/fbioe.2020.00990 32903562 PMC7438450

[B37] Huang N. Liu Y. Fang Y. Zheng S. Wu J. Wang M. (2020). Gold Nanoparticles Induce Tumor Vessel Normalization and Impair Metastasis by Inhibiting Endothelial Smad2/3 Signaling. ACS Nano 14 (7), 7940–7958. Epub 2020 Jun 15. PMID: 32413258. 10.1021/acsnano.9b08460 32413258

[B38] Huang X. Qian W. El-Sayed I. H. El-Sayed M. A. (2007). The Potential Use of the Enhanced Nonlinear Properties of Gold Nanospheres in Photothermal Cancer Therapy. Lasers Surg. Med. 39, 747–753. 10.1002/lsm.20577 17960762

[B39] Huang Y. Goel S. Duda D. G. Fukumura D. Jain R. K. (2013). Vascular Normalization as an Emerging Strategy to Enhance Cancer Immunotherapy. Cancer Res. 73 (10), 2943–2948. Epub 2013 Feb 25. PMID: 23440426; PMCID: PMC3655127. 10.1158/0008-5472.CAN-12-4354 23440426 PMC3655127

[B40] Huang Y. Kim B. Y. S. Chan C. K. Hahn S. M. Weissman I. L. Jiang W. (2018). Improving Immune-Vascular Crosstalk for Cancer Immunotherapy. Nat. Rev. Immunol. 18, 195–203. 10.1038/nri.2017.145 29332937 PMC5922422

[B41] Ibrahim K. E. Bakhiet A. O. Awadalla M. E. Khan H. A. (2018). A Priming Dose Protects against Gold Nanoparticles-Induced Proinflammatory Cytokines mRNA Expression in Mice. Nanomedicine (Lond) 13 (3), 313–323. Epub 2017 Dec 12. PMID: 29231780. 10.2217/nnm-2017-0332 29231780

[B42] Imanparast A. Bakhshizadeh M. Salek R. Sazgarnia A. (2018). Pegylated Hollow Gold-Mitoxantrone Nanoparticles Combining Photodynamic Therapy and Chemotherapy of Cancer Cells. Photodiagnosis Photodyn Ther. 23, 295–305. 10.1016/j.pdpdt.2018.07.011 30048763

[B43] Jain R. K. (2013). Normalizing Tumor Microenvironment to Treat Cancer: Bench to Bedside to Biomarkers. J. Clin. Oncol. 31 (17), 2205–2218. Epub 2013 May 13. PMID: 23669226; PMCID: PMC3731977. 10.1200/JCO.2012.46.3653 23669226 PMC3731977

[B44] Jain S. Coulter J. A. Hounsell A. R. Butterworth K. T. McMahon S. J. Hyland W. B. (2011). Cell-specific Radiosensitization by Gold Nanoparticles at Megavoltage Radiation Energies. Int. J. Radiat. Oncol. Biol. Phys. 79, 531–539. 10.1016/j.ijrobp.2010.08.044 21095075 PMC3015172

[B45] Jeynes J. C. Merchant M. J. Spindler A. Wera A. C. Kirkby K. J. (2014). Investigation of Gold Nanoparticle Radiosensitization Mechanisms Using a Free Radical Scavenger and Protons of Different Energies. Phys. Med. Biol. 59, 6431–6443. 10.1088/0031-9155/59/21/6431 25296027

[B46] Kang T. Huang Y. Zhu Q. Cheng H. Pei Y. Feng J. (2018). Necroptotic Cancer Cells-Mimicry Nanovaccine Boosts Anti-tumor Immunity with Tailored Immune-Stimulatory Modality. Biomaterials 164, 80–97. 10.1016/j.biomaterials.2018.02.033 29499438

[B47] Khalil D. N. Smith E. L. Brentjens R. J. Wolchok J. D. (2016). The Future of Cancer Treatment: Immunomodulation, CARs and Combination Immunotherapy. Nat. Rev. Clin. Oncol. 13, 394. 10.1038/nrclinonc.2016.65 27118494 PMC5558237

[B48] Klębowski B. Depciuch J. Parlińska-Wojtan M. Baran J. (2018). Applications of noble Metal-Based Nanoparticles in Medicine. Ijms 19, 4031. 10.3390/ijms19124031 PMC6320918 30551592

[B49] Krishna Priya S. Nagare R. P. Sneha V. S. Sidhanth C. Bindhya S. Manasa P. (2016). Tumour Angiogenesis-Origin of Blood Vessels. Int. J. Cancer 139, 729–735. 10.1002/ijc.30067 26934471

[B50] Labala S. Jose A. Chawla S. R. Khan M. S. Bhatnagar S. Kulkarni O. P. (2017). Effective Melanoma Cancer Suppression by Iontophoretic Co-delivery of STAT3 siRNA and Imatinib Using Gold Nanoparticles. Int. J. Pharm. 525, 407–417. 10.1016/j.ijpharm.2017.03.087 28373100

[B51] Lakshminarayanan R. Ye E. Young D. J. Li Z. Loh X. J. (2018). Recent Advances in the Development of Antimicrobial Nanoparticles for Combating Resistant Pathogens. Adv. Healthc. Mater. 7, e1701400. 10.1002/adhm.201701400 29717819 PMC7161883

[B52] Lee J. H. Choi J. W. (2018). Application of Plasmonic Gold Nanoparticle for Drug Delivery System. Curr. Drug Targets 19, 271–278. 10.2174/1389450118666170427150257 28460609

[B53] Li B. Chan H. L. Chen P. (2019). Immune Checkpoint Inhibitors: Basics and Challenges. Curr. Med. Chem. 26, 3009–3025. 10.2174/0929867324666170804143706 28782469

[B54] Li S. Yang Y. Lin X. Li Z. Ma G. Su Z. (2020). Biocompatible Cationic Solid Lipid Nanoparticles as Adjuvants Effectively Improve Humoral and T Cell Immune Response of Foot and Mouth Disease Vaccines. Vaccine 38, 2478–2486. 10.1016/j.vaccine.2020.02.004 32057580

[B55] Li W. Li X. Liu S. Yang W. Pan F. Yang X. Y. (2017). Gold Nanoparticles Attenuate Metastasis by Tumor Vasculature Normalization and Epithelial-Mesenchymal Transition Inhibition. Int. J. Nanomedicine 12, 3509–3520. PMID: 28496326; PMCID: PMC5422535. 10.2147/IJN.S128802 28496326 PMC5422535

[B56] Liang R. Liu L. He H. Chen Z. Han Z. Luo Z. (2018). Oxygen-boosted Immunogenic Photodynamic Therapy with Gold Nanocages@manganese Dioxide to Inhibit Tumor Growth and Metastases. Biomaterials 177, 149–160. 10.1016/j.biomaterials.2018.05.051 29890364

[B57] Libutti S. K. Paciotti G. F. Byrnes A. A. Alexander H. R. Gannon W. E. Walker M. (2010). Phase I and Pharmacokinetic Studies of CYT-6091, a Novel PEGylated Colloidal Gold-rhTNF Nanomedicine. Clin. Cancer Res. 16, 6139–6149. 10.1158/1078-0432.Ccr-10-0978 20876255 PMC3004980

[B58] Liu B. Cao W. Cheng J. Fan S. Pan S. Wang L. (2019). Human Natural Killer Cells for Targeting Delivery of Gold Nanostars and Bimodal Imaging Directed Photothermal/photodynamic Therapy and Immunotherapy. Cancer Biol. Med. 16, 756–770. 10.20892/j.issn.2095-3941.2019.0112 31908893 PMC6936231

[B59] Liu B. Cao W. Qiao G. Yao S. Pan S. Wang L. (2019). Effects of Gold Nanoprism-Assisted Human PD-L1 siRNA on Both Gene Down-Regulation and Photothermal Therapy on Lung Cancer. Acta Biomater. 99, 307–319. Epub 2019 Sep 9. PMID: 31513911. 10.1016/j.actbio.2019.08.046 31513911

[B60] Liu B. Cao W. Qiao G. Yao S. Pan S. Wang L. (2019). Effects of Gold Nanoprism-Assisted Human PD-L1 siRNA on Both Gene Down-Regulation and Photothermal Therapy on Lung Cancer. Acta Biomater. 99, 307–319. 10.1016/j.actbio.2019.08.046 31513911

[B61] Liu J. Yang S. Cao B. Zhou G. Zhang F. Wang Y. (2021). Targeting B7-H3 via Chimeric Antigen Receptor T Cells and Bispecific Killer Cell Engagers Augments Antitumor Response of Cytotoxic Lymphocytes. J. Hematol. Oncol. 14, 21. 10.1186/s13045-020-01024-8 33514401 PMC7844995

[B62] Liu Y. Crawford B. M. Vo-Dinh T. (2018). Gold Nanoparticles-Mediated Photothermal Therapy and Immunotherapy. Immunotherapy 10, 1175–1188. 10.2217/imt-2018-0029 30236026

[B63] Lopez-Campos F. Candini D. Carrasco E. Berenguer Francés M. A. (2019). Nanoparticles Applied to Cancer Immunoregulation. Rep. Pract. Oncol. Radiother. 24, 47–55. 10.1016/j.rpor.2018.10.001 30425606 PMC6223232

[B64] Luo L. Zhu C. Yin H. Jiang M. Zhang J. Qin B. (2018). Laser Immunotherapy in Combination with Perdurable PD-1 Blocking for the Treatment of Metastatic Tumors. ACS Nano 12, 7647–7662. 10.1021/acsnano.8b00204 30020768

[B65] Mahmoodi Chalbatani G. Dana H. Gharagouzloo E. Grijalvo S. Eritja R. Logsdon C. D. (2019). Small Interfering RNAs (siRNAs) in Cancer Therapy: A Nano-Based Approach. Int. J. Nanomedicine 14, 3111–3128. 10.2147/ijn.S200253 31118626 PMC6504672

[B66] Maity A. Pore N. Lee J. Solomon D. O'Rourke D. M. (2000). Epidermal Growth Factor Receptor Transcriptionally Up-Regulates Vascular Endothelial Growth Factor Expression in Human Glioblastoma Cells via a Pathway Involving Phosphatidylinositol 3'-kinase and Distinct from that Induced by Hypoxia. Cancer Res. 60, 5879–5886. 11059786

[B67] Mattheolabakis G. Mikelis C. M. (2019). Nanoparticle Delivery and Tumor Vascular Normalization: The Chicken or the Egg?. Front. Oncol. 9, 1227. 10.3389/fonc.2019.01227 31799190 PMC6863425

[B68] Mazzieri R. Pucci F. Moi D. Zonari E. Ranghetti A. Berti A. (2011). Targeting the ANG2/TIE2 axis Inhibits Tumor Growth and Metastasis by Impairing Angiogenesis and Disabling Rebounds of Proangiogenic Myeloid Cells. Cancer Cell 19 (4), 512–526. PMID: 21481792. 10.1016/j.ccr.2011.02.005 21481792

[B69] McMahon S. J. Hyland W. B. Muir M. F. Coulter J. A. Jain S. Butterworth K. T. (2011). Nanodosimetric Effects of Gold Nanoparticles in Megavoltage Radiation Therapy. Radiother. Oncol. 100, 412–416. 10.1016/j.radonc.2011.08.026 21924786

[B70] Meir R. Shamalov K. Sadan T. Motiei M. Yaari G. Cohen C. J. (2017). Fast Image-Guided Stratification Using Anti-programmed Death Ligand 1 Gold Nanoparticles for Cancer Immunotherapy. ACS Nano 11, 11127–11134. 10.1021/acsnano.7b05299 29028305

[B71] Menon S. Shin S. Dy G. (2016). Advances in Cancer Immunotherapy in Solid Tumors. Cancers (Basel) 8, 106. 10.3390/cancers8120106 PMC5187504 27886124

[B72] Mioc A. Mioc M. Ghiulai R. Voicu M. Racoviceanu R. Trandafirescu C. (2019). Gold Nanoparticles as Targeted Delivery Systems and Theranostic Agents in Cancer Therapy. Curr. Med. Chem. 26, 6493–6513. 10.2174/0929867326666190506123721 31057102

[B73] Ou Y. C. Wen X. Bardhan R. (2020). Cancer Immunoimaging with Smart Nanoparticles. Trends Biotechnol. 38, 388–403. 10.1016/j.tibtech.2019.11.001 31812371

[B74] Overchuk M. Zheng G. (2018). Overcoming Obstacles in the Tumor Microenvironment: Recent Advancements in Nanoparticle Delivery for Cancer Theranostics. Biomaterials 156, 217–237. Epub 2017 Oct 20. PMID: 29207323. 10.1016/j.biomaterials.2017.10.024 29207323

[B75] Paciotti G. F. Kingston D. G. I. Tamarkin L. (2006). Colloidal Gold Nanoparticles: A Novel Nanoparticle Platform for Developing Multifunctional Tumor-Targeted Drug Delivery Vectors. Drug Dev. Res. 67, 47–54. 10.1002/ddr.20066

[B76] Pan Y. Ding H. Qin L. Zhao X. Cai J. Du B. (2013). Gold Nanoparticles Induce Nanostructural Reorganization of VEGFR2 to Repress Angiogenesis. J. Biomed. Nanotechnol 9, 1746–1756. 10.1166/jbn.2013.1678 24015504

[B77] Pietro P. D. Strano G. Zuccarello L. Satriano C. (2016). Gold and Silver Nanoparticles for Applications in Theranostics. Curr. Top. Med. Chem. 16, 3069–3102. 10.2174/1568026616666160715163346 27426869

[B78] Popescu R. C. Grumezescu A. M. (2015). Metal Based Frameworks for Drug Delivery Systems. Curr. Top. Med. Chem. 15, 1532–1542. 10.2174/1568026615666150414145323 25877086

[B79] Qin S. Xu L. Yi M. Yu S. Wu K. Luo S. (2019). Novel Immune Checkpoint Targets: Moving beyond PD-1 and CTLA-4. Mol. Cancer 18, 155. 10.1186/s12943-019-1091-2 31690319 PMC6833286

[B80] Qiu H. Min Y. Rodgers Z. Zhang L. Wang A. Z. (2017). Nanomedicine Approaches to Improve Cancer Immunotherapy. Wiley Interdiscip Rev Nanomed Nanobiotechnol, 9. 10.1002/wnan.1456 PMC5561449 28296286

[B81] Qiu T. A. Bozich J. S. Lohse S. E. Vartanian A. M. Jacob L. M. Meyer B. M. (2015). Gene Expression as an Indicator of the Molecular Response and Toxicity in the Bacterium Shewanella Oneidensis and the Water Flea Daphnia magna Exposed to Functionalized Gold Nanoparticles. Environ. Sci. Nano 2, 615–629. 10.1039/C5EN00037H

[B82] Rajendrakumar S. K. Uthaman S. Cho C. S. Park I. K. (2018). Nanoparticle-Based Phototriggered Cancer Immunotherapy and its Domino Effect in the Tumor Microenvironment. Biomacromolecules 19 (6), 1869–1887. Epub 2018 Apr 27. PMID: 29677439. 10.1021/acs.biomac.8b00460 29677439

[B83] Retif P. Pinel S. Toussaint M. Frochot C. Chouikrat R. Bastogne T. (2015). Nanoparticles for Radiation Therapy Enhancement: The Key Parameters. Theranostics 5, 1030–1044. 10.7150/thno.11642 26155318 PMC4493540

[B84] Riley R. S. Day E. S. (2017). Gold Nanoparticle-Mediated Photothermal Therapy: Applications and Opportunities for Multimodal Cancer Treatment. Wiley Interdiscip Rev Nanomed Nanobiotechnol, 9. 10.1002/wnan.1449 PMC5474189 28160445

[B85] Riley R. S. June C. H. Langer R. Mitchell M. J. (2019). Delivery Technologies for Cancer Immunotherapy. Nat. Rev. Drug Discov. 18, 175–196. 10.1038/s41573-018-0006-z 30622344 PMC6410566

[B86] Ruan S. Xie R. Qin L. Yu M. Xiao W. Hu C. (2019). Aggregable Nanoparticles-Enabled Chemotherapy and Autophagy Inhibition Combined with Anti-PD-L1 Antibody for Improved Glioma Treatment. Nano Lett. 19, 8318–8332. 10.1021/acs.nanolett.9b03968 31610656

[B87] Savitsky K. Yu X. (2019). Combined Strategies for Tumor Immunotherapy with Nanoparticles. Clin. Transl Oncol. 21, 1441–1449. 10.1007/s12094-019-02081-3 31055713

[B88] Sehgal K. Dhodapkar K. M. Dhodapkar M. V. (2014). Targeting Human Dendritic Cells *In Situ* to Improve Vaccines. Immunol. Lett. 162, 59–67. 10.1016/j.imlet.2014.07.004 25072116 PMC4506641

[B89] Shinchi H. Yamaguchi T. Moroishi T. Yuki M. Wakao M. Cottam H. B. (2019). Gold Nanoparticles Coimmobilized with Small Molecule Toll-like Receptor 7 Ligand and α-Mannose as Adjuvants. Bioconjug. Chem. 30, 2811–2821. 10.1021/acs.bioconjchem.9b00560 31560198

[B90] Singh P. Pandit S. Mokkapati V. R. S. S. Garg A. Ravikumar V. Mijakovic I. (2018). Gold Nanoparticles in Diagnostics and Therapeutics for Human Cancer. Int. J. Mol. Sci. 19, 1979. 10.3390/ijms19071979 PMC6073740 29986450

[B91] Surendran S. P. Moon M. J. Park R. Jeong Y. Y. (2018). Bioactive Nanoparticles for Cancer Immunotherapy. Ijms 19, 3877. 10.3390/ijms19123877 PMC6321368 30518139

[B92] Sztandera K. Gorzkiewicz M. Klajnert-Maculewicz B. (2019). Gold Nanoparticles in Cancer Treatment. Mol. Pharm. 16, 1–23. 10.1021/acs.molpharmaceut.8b00810 30452861

[B93] Tan S. Li D. Zhu X. (2020). Cancer Immunotherapy: Pros, Cons and beyond. Biomed. Pharmacother. 124, 109821. 10.1016/j.biopha.2020.109821 31962285

[B94] Tomić S. Ðokić J. Vasilijić S. Ogrinc N. Rudolf R. Pelicon P. (2014). Size-dependent Effects of Gold Nanoparticles Uptake on Maturation and Antitumor Functions of Human Dendritic Cells *In Vitro* . PLoS One 9, e96584. 10.1371/journal.pone.0096584 24802102 PMC4011871

[B95] Tshikhudo T. R. Wang Z. Brust M. (2004). Biocompatible Gold Nanoparticles. Mater. Sci. Tech. 20, 980–984. 10.1179/026708304225019849

[B96] Wang Y. Zhou S. Yang F. Qi X. Wang X. Guan X. (2019). Treatment-related Adverse Events of PD-1 and PD-L1 Inhibitors in Clinical Trials: A Systematic Review and Meta-Analysis. JAMA Oncol. 5, 1008–1019. 10.1001/jamaoncol.2019.0393 31021376 PMC6487913

[B97] Weintraub K. (2013). Biomedicine: The New Gold Standard. Nature 495, S14–S16. 10.1038/495S14a 23486097

[B98] Xia L. Liu Y. Wang Y. (2019). PD-1/PD-L1 Blockade Therapy in Advanced Non-small-cell Lung Cancer: Current Status and Future Directions. Oncologist 24, S31–s41. 10.1634/theoncologist.2019-IO-S1-s05 30819829 PMC6394772

[B99] Xue X. Li J. Fan Y. (2021). Gene Silencing-Mediated Immune Checkpoint Blockade for Tumor Therapy Boosted by Dendrimer-Entrapped Gold Nanoparticles. Sci. China Mater. 10.1007/s40843-020-1591-1

[B100] Yang M. Li J. Gu P. Fan X. (2021). The Application of Nanoparticles in Cancer Immunotherapy: Targeting Tumor Microenvironment. Bioact Mater. 6, 1973–1987. 10.1016/j.bioactmat.2020.12.010 33426371 PMC7773537

[B101] Yin H. Kanasty R. L. Eltoukhy A. A. Vegas A. J. Dorkin J. R. Anderson D. G. (2014). Non-viral Vectors for Gene-Based Therapy. Nat. Rev. Genet. 15, 541–555. 10.1038/nrg3763 25022906

[B102] Yu H. J. De Geest B. G. (2020). Nanomedicine and Cancer Immunotherapy. Acta Pharmacol. Sin 41, 879–880. 10.1038/s41401-020-0426-2 32467567 PMC7471393

[B103] Yu W. D. Sun G. Li J. Xu J. Wang X. (2019). Mechanisms and Therapeutic Potentials of Cancer Immunotherapy in Combination with Radiotherapy And/or Chemotherapy. Cancer Lett. 452, 66–70. 10.1016/j.canlet.2019.02.048 30902563

[B104] Zhang D. Wu T. Qin X. Qiao Q. Shang L. Song Q. (2019). Intracellularly Generated Immunological Gold Nanoparticles for Combinatorial Photothermal Therapy and Immunotherapy against Tumor. Nano Lett. 19, 6635–6646. 10.1021/acs.nanolett.9b02903 31393134

[B105] Zhang Y. Xiong X. Huai Y. Dey A. Hossen M. N. Roy R. V. (2019). Gold Nanoparticles Disrupt Tumor Microenvironment - Endothelial Cell Cross Talk to Inhibit Angiogenic Phenotypes *In Vitro* . Bioconjug. Chem. 30, 1724–1733. 10.1021/acs.bioconjchem.9b00262 31067032 PMC6939887

[B106] Zhao Z. Zheng L. Chen W. Weng W. Song J. Ji J. (2019). Delivery Strategies of Cancer Immunotherapy: Recent Advances and Future Perspectives. J. Hematol. Oncol. 12, 126. 10.1186/s13045-019-0817-3 31779642 PMC6883629

[B107] Zhou F. Nordquist R. E. Chen W. R. (2016). Photonics Immunotherapy - A Novel Strategy for Cancer Treatment. J. Innov. Opt. Health Sci. 09, 1630001. 10.1142/S1793545816300019

[B108] Zhou X. Liu R. Qin S. Yu R. Fu Y. (2016). Current Status and Future Directions of Nanoparticulate Strategy for Cancer Immunotherapy. Curr. Drug Metab. 17, 755–762. 10.2174/1389200217666160714095722 27411558

